# Higher blood cotinine level is associated with worse cognitive functioning in non-smoking older adults

**DOI:** 10.3389/fnins.2022.1080066

**Published:** 2022-11-24

**Authors:** Zhenmei Fu, Guiye Qi, Zhe Qu, Xuechun Lin, Li Xu, Biyu Shen, Fanghong Dong, Song Ge

**Affiliations:** ^1^Department of Radiology, Shandong Provincial Hospital Affiliated to Shandong First Medical University, Jinan, Shandong, China; ^2^Department of Medical Engineering, Shandong Provincial Hospital Affiliated to Shandong First Medical University, Jinan, Shandong, China; ^3^School of Nursing, Xuzhou Medical University, Xuzhou, Jiangsu, China; ^4^Department of Nutrition and Food Hygiene, Hubei Key Laboratory of Food Nutrition and Safety, Ministry of Education Key Laboratory of Environment and Health, School of Public Health, Tongji Medical College, Huazhong University of Science and Technology, Wuhan, China; ^5^Department of Pulmonary and Critical Care Medicine, Shandong Provincial Hospital Affiliated to Shandong First Medical University, Jinan, Shandong, China; ^6^Department of Nursing, Shanghai Children’s Medical Center Affiliated to Shanghai Jiao Tong University School of Medicine, Shanghai, China; ^7^School of Nursing, Hebei University, Baoding, China; ^8^Department of Natural Sciences, University of Houston-Downtown, Houston, TX, United States

**Keywords:** cotinine, cognitive function, older adults, NHANES, secondhand smoke, tobacco

## Abstract

**Introduction:**

Secondhand smoke (SHS) is common in older adults; however, its cognitive effect is unclear. We aimed to examine the association between serum cotinine level and cognitive functioning among non-smoking older adults.

**Materials and methods:**

A total of 2,703 older adults aged 60 and above from the National Health and Nutrition Examination (NHANES) Survey 2011–2014 were included. Serum cotinine level was analyzed in the laboratory. A level ≤10 ng/ml and a response of “no” to the question “Do you currently smoke?” were used to select non-smokers. Cognitive functioning was measured using the Consortium to Establish a Registry for Alzheimer’s disease Word Learning subtest (CERAD-WL) immediate and delayed recall tests, the Animal Fluency test (AFT), and the Digit Symbol Substitution test (DSST). Multivariable linear regression models were constructed to examine the association between serum cotinine level quartile and test-specific and global cognition *z* scores adjusting for age, race/ethnicity, education, depressive symptoms, body mass index, alcohol use, smoking history, prevalent coronary heart disease (CHD), stroke, and systolic blood pressure.

**Results:**

About half of the participants (mean age 70.5 years) were female (53.6%), non-Hispanic White (48.3%), and completed some college and above (50.2%). Multivariate linear regressions with a reference group being those in the 1st quantile (lowest) showed that participants in the 4th quartile (highest) of serum cotinine level had lower immediate recall [β = −0.16, 95% confidence interval (CI) = −0.29, −0.03], AFT (β = −0.19, 95% CI = −0.33, −0.05), DSST (β = −0.27, 95% CI = −0.39, −0.15), and global cognition (β = −0.26, 95% CI = −0.39, −0.14) *z* scores. Participants in the 3rd quartile had lower immediate recall (β = −0.16, 95% CI = −0.30, −0.02) and global cognition (β = −0.16, 95% CI = −0.29, −0.02) *z* scores. Participants in the 2nd quartile had lower delayed recall *z* scores (β = −0.16, 95% CI = −0.29, −0.02).

**Conclusion:**

Higher serum cotinine level was associated with worse cognitive functioning in non-smoking older adults. Prevention and reduction of SHS in older adults may help protect their cognitive functioning.

## Introduction

Dementia primarily affects older adults, with an age-standardized prevalence ranging from 5 to 7% in older adults aged over 60 in most parts of the world (Prince et al., 2013). With 33.1 million disability-adjusted life years lost in 2019, Alzheimer’s disease and related dementia (ADRD) has emerged as a major global cause of mortality and morbidity in older adults (Nandi et al., 2022). With the population aging, by 2060, 13.9 million Americans are expected to have ADRD, with the prevalence of ADRD reaching 3.3% in US adults aged ≥65 years (Matthews et al., 2019). Globally, 35.6 million people were estimated to have dementia in 2010, which is expected to nearly double in 20 years, reaching 65.7 million in 2030 and 115.4 million in 2050 (Prince et al., 2013). Thus, older adults, their caregivers, communities, and healthcare systems worldwide are seriously threatened by ADRD now and in the future. ADRD negatively affects a person’s memory, thought processes, and functioning (Ge et al., 2018, 2020). People with ADRD and their families are subject to a significant financial burden as a result, which is primarily incurred by increased home healthcare costs (Deb et al., 2018). Since ADRD is currently incurable, one of the main strategies to reduce the burden of the disease is to spot and address modifiable risk factors before the clinical manifestation of ADRD.

Active smoking is considered a modifiable risk factor for ADRD (Peters et al., 2008; Rusanen et al., 2011). Even though cigarette smoking has declined in the US over the past few decades, it is still common among older adults, with 9% of those 65 and older reporting that they currently smoke (Cornelius et al., 2022). Many studies have examined the relationship between active tobacco smoking and cognitive functioning in older adults, with most studies supporting the harmful cognitive effects of tobacco smoking (Ott et al., 2004; Llewellyn et al., 2009; Orsitto et al., 2012; Vermeulen et al., 2018; Nadar et al., 2021) and a few having the opposite finding (Ge et al., 2020). Besides active smoking, secondhand smoke (SHS) exposes a lot of older adults to tobacco (Craciun et al., 2022). SHS, also called passive smoking, refers to a circumstance in which a non-smoker is exposed to either side-stream or mainstream smoke and therefore inhales another person’s smoke (Ling and Heffernan, 2016). A limited number of studies have examined the effect of SHS on cognitive functioning in this population (Llewellyn et al., 2009). Among the existing studies, most used participants’ self-report exposure to SHS (Friedrich, 2007; Barnes et al., 2010; Heffernan and O’Neill, 2013; Bai et al., 2020) and thus was unprecise and subject to recall and report bias. Thus, studies involving the use of biomarkers to objectively measure the extent of SHS are needed to clarify its relationship with cognitive functioning in this growing population.

In this study, we utilized the National Health and Nutrition Examination Study (NHANES) 2011–2014 waive of data (NHANES) to examine the relationship between serum cotinine level and cognitive functioning in a group of non-smoking older adults. The findings of this study will provide implications for clinical practice and policy development to protect cognitive functioning in the growing number of older adults in most countries of the world.

## Materials and methods

### The parent study design and recruitment

Every 2 years, the NHANES, a continuous cross-sectional survey of civilian, non-institutionalized adults and children in the United States, is conducted by the National Center for Health Statistics of the Centers for Disease Control and Prevention (CDC) (NHANES). A complex, multistage probability strategy involving a selection of census blocks or area segments within clusters of census blocks is used to recruit participants all over the US for each 2-year cycle (Johnson et al., 2013). The socio-demographic, health, and nutritional status of participants are evaluated using in-person interviews at participants’ homes and medical exams at mobile exam centers with specialized equipment. For this analysis, the NHANES 2011–2012 and the NHANES (2013/2014) were merged to increase power. People who were aged 60 and above, did not actively smoke at the time of the survey, and had available information on serum cotinine level and cognitive functioning were included. A total of 9,338 individuals took part in the NHANES 2011–2012 survey and 9,813 individuals in the NHANES (2013/2014) survey. We excluded those who aged < 60 (*n* = 15,679), had missing data on serum cotinine level (*n* = 228), self-reported “yes” to the question, “Do you currently smoke?” (*n* = 423), or had a serum cotinine level > 10 ng/ml (*n* = 118). People whose serum cotinine level > 10 ng/ml were excluded from the analysis because serum cotinine level greater than 10 ng/ml is almost universally present in every smoker (Hukkanen et al., 2005). Finally, a total of 2,703 non-smoking participants aged 60 and above were included in this study.

### Ethical considerations

The National Center for Health Statistics Research Ethics Review Board gave its approval to NHANES. This study was granted an exemption by the University of Houston-Downtown Committee for the Protection of Human Subjects because we only used de-identified, publicly available data.

### Measures

#### Independent variable: Quartile of serum cotinine level (ng/ml)

As the primary metabolite of nicotine with a half-life of about 15–20 h, cotinine levels in bodily fluids can serve as indicators of SHS as well as active smoking (Llewellyn et al., 2009). The Division of Laboratory Sciences, National Center for Environmental Health, and CDC collected participants’ serum samples during physical exams, aliquoted them, and kept them frozen at −20°C until they could be analyzed. The isotope-dilution high-performance liquid chromatography/atmospheric pressure chemical ionization tandem mass spectrometric (ID HPLC-APCI MS/MS) technique was used to measure serum cotinine level. The detailed method has been published elsewhere (Jacob et al., 2011). A blank and two quality-control pools were used in each analytical run. Quality control/quality assurance program was conducted by the Division of Laboratory Sciences, National Center for Environmental Health, and CDC; therefore, precise and accurate results were reported (Caudill et al., 2008). Using the variance from the repeated analysis of a small, spiked serum sample (0.2 ml), this method for measuring serum cotinine has a lower detection limit of 0.015 ng/ml.

#### Dependent variable: Cognitive functioning

Several cognitive psychomotor tests, including the Consortium to Establish a Registry for Alzheimer’s Disease Word Learning subtest (CERAD-WL), the Animal Fluency test (AFT), and the Digit Symbol Substitution test (DSST) were used to assess participants’ cognitive functioning.

(1) The CERAD-WL assessed participants’ immediate and delayed learning ability for new verbal information and included an immediate recall test and a delayed recall test after three successive immediate learning trials (Davis et al., 1992; Fillenbaum et al., 2008). In the immediate recall test, participants were required to read aloud ten random words displayed on a computer screen as large, bolded letters, one at a time, during each of the three learning trials. Participants were instructed to remember and recall as many of the 10 words as they could right away. Each time, these 10 words were presented in a different order. The maximum score for each trial is 10. Consequently, the participant’s immediate recall score was represented by the sum of the three trials’ scores, which ranged from 0 to 30. Participants were asked to recall as many words from the same 10-word list as they could after completing the DSST and AFT, the other two cognitive tests. This served as their delayed recall test. The number of accurate words that the participant could recall determined their delayed recall test score, which ranged from 0 to 10. It has been used in major epidemiologic studies of diverse racial and cultural communities (Morris et al., 1989; Prince et al., 2003; Gao et al., 2009).

(2) The AFT was used to measure participants’ language fluency, a component of executive function (Strauss et al., 2006). The participants were given 60 s to name as many animals as they could. One point was awarded for each animal identified. The AFT has been shown to differentiate mild cognitive impairment and probable Alzheimer’s disease in older people with a sensitivity of 98.8% (García-Herranz et al., 2020). The AFT has been used in large-scale screenings and epidemiologic studies (Clark et al., 2009).

(3) Participants’ processing speed, sustained attention, and working memory was evaluated by the DSST, which was a performance module from the Wechsler Adult Intelligence Scale (WAIS-III) (Ryan and Schnakenberg-Ott, 2003). This test was given using a paper form with a top-mounted key that had nine numbers and paired symbols. The 133 boxes next to the 133 numbers containing the corresponding symbols had to have the symptoms copied to them within the allotted 2 min. The total number of right matches determined the DSST score, ranging between 0 and 133. The DSST has been used in large screening, epidemiological, and clinical studies (Plassman et al., 2007; Proust-Lima et al., 2007).

### Covariates

To control for potential confounding between serum cotinine level and cognitive functioning, after reviewing several related studies (Peters et al., 2008; Pan et al., 2018; Ge et al., 2020; Yang et al., 2022), we included the following covariates in the analysis- age (years), sex (male or female), race/ethnicity (Mexican Americans, other Hispanics, non-Hispanic White, or non-Hispanic Black), education (below high school, high school graduate, or some college or above), depressive symptoms, body mass index (<18.5, 18.5–24.9, 25–29.9, or ≥30 kg/m^2^), alcohol use (0–1 drink per day, 2 drinks per day, or 3 and more drinks per day), smoking history (former smokers or never smokers), prevalent coronary heart disease (CHD) (yes or no), stroke (yes or no), and systolic blood pressure (mmHg). All the above information was collected from face-to-face interviews or assessed during health exams. The Patient Health Questionnaire (PHQ-9) total score (range 0–27) was used to represent depressive symptoms (Kroenke et al., 2001). Regarding smoking history, if participants responded “no” to the question, “Have you smoked at least 100 cigarettes in your entire life?,” they were categorized as never smokers; otherwise, they were categorized as former smokers.

### Statistical analysis

Standardized *z* scores of the CERAD-WL immediate recall, the CERAD-WL delayed recall, the AFT, and the DSST were calculated using means and standard deviations of the cognitive test scores. Then, global cognition *z*-scores were calculated using the means and standard deviations of all test-specific *z*-scores. Multivariable regression models were used to examine the independent relationship between quartiles of serum cotinine level (reference: 4th quartile, the lowest quantile) and test-specific and global cognition *z*-scores, controlling the covariates mentioned above. Prior to constructing the regression models, we examined whether there was multicollinearity among the covariates. The variance inflation factor (VIF) was less than 10, indicating no multicollinearity (Miles, 2014). A 95% confidence interval (CI) excluding zero was considered as statistical significance. All analyses were performed using SPSS 25.0.

## Results

The characteristics of the excluded participants due to missing values and being current smokers (*n* = 769) were summarized in the Supplementary Appendix. Compared with the included participants, the excluded participants were younger, more likely to be male, non-Hispanic Blacks, and completed less education. They were also more likely to have higher systolic blood pressure, more depressive symptoms, normal body mass index, more alcohol use, lower CERAD W-L delayed recall, lower AFT, and lower DSST scores.

The characteristics of the study population were presented in Table 1. The 2,703 participants had a mean age of 70.5 years [standard deviation (SD) = 7.0]. About half of them were female (53.6%), non-Hispanic White (48.3%), completed some college or above (50.2%), had a BMI ≥ 30 kg/m^2^ (38.1%), drank 0–1 drink of alcohol use per day (29.3%), and were never smokers (58.2%). The participants had a mean of 2.4 h of physical activity every week, a mean of 190.4 mg/dl total cholesterol, and a mean of 124.5 mmHg systolic blood pressure. Their mean serum cotinine level (ng/ml) was 0.13 (SD = 0.60), ranging from 0.01 to 9.90. Their mean CERAD-WL immediate recall, CERAD-WL delayed recall, AFT, and DSST score was 18.6 (SD = 5.1), 5.8 (SD = 2.4), 16.5 (SD = 5.5), and 46.5 (SD = 17.5), respectively.

**TABLE 1:** Characteristics of the participants by quartile of serum cotinine level^a^.

Variables	Quartile 1 ≤0.01 ng/ml (*n* = 1,205)	Quartile 2 0.01 < cotinine ≤ 0.02 ng/ml (*n* = 451)	Quartile 3 0.02 < cotinine ≤ 0.04 ng/ml (*n* = 398)	Quartile 4 >0.04 ng/ml (*n* = 649)	Total (*n* = 2,703)
Age (years)	71.4 (6.9)	69.9 (7.0)	69.5 (6.8)	69.7 (7.0)	70.5 (7.0)
**Sex [*n* (%)]**					
Male	517 (42.9%)	216 (47.9%)	189 (47.5%)	331 (51.0%)	1,253 (46.4%)
Female	688 (57.1%)	235 (52.1%)	209 (52.5%)	318 (49.0%)	1,450 (53.6%)
**Race/ethnicity [*n* (%)]**					
Mexican Americans	123 (10.2%)	49 (10.9%)	28 (7.0%)	49 (7.6%)	249 (9.2%)
Other Hispanics	123 (10.2%)	55 (12.2%)	50 (12.6%)	43 (6.6%)	271 (10.0%)
Non-Hispanic Whites	717 (59.5%)	182 (40.4%)	163 (41.0%)	243 (37.4%)	1,305 (48.3%)
Non-Hispanic Blacks	150 (12.4%)	98 (21.7%)	87 (21.9%)	223 (34.4%)	558 (20.6%)
Other	92 (7.6%)	67 (14.9%)	70 (17.6%)	91 (14.0%)	320 (11.8%)
**Education [*n* (%)]**					
Below high school	256 (21.3%)	133 (29.5%)	112 (28.1%)	222 (34.2%)	723 (26.7%)
High school graduate	249 (20.7%)	96 (21.3%)	106 (26.6%)	170 (26.2%)	621 (23.0%)
Some college or above	699 (58.0%)	222 (49.2%)	179 (45.0%)	255 (39.3%)	1,355 (50.2%)
**Body mass index [*n* (%)]**					
<18.5 kg/m^2^	12 (1.0%)	5 (1.1%)	3 (0.8%)	11 (1.7%)	31 (1.1%)
18.5–24.9 kg/m^2^	300 (24.9%)	104 (23.1%)	96 (24.1%)	137 (21.1%)	637 (23.6%)
25.0–29.9 kg/m^2^	429 (35.6%)	167 (37.0%)	146 (36.7%)	214 (33.0%)	956 (35.4%)
≥30 kg/m^2^	445 (36.9%)	167 (37.0%)	143 (35.9%)	274 (42.2%)	1,029 (38.1%)
**Smoking**					
Never	745 (61.8%)	277 (61.4%)	233 (58.5%)	318 (49.0%)	1,573 (58.2%)
Former	460 (38.2%)	173 (38.4%)	165 (41.5%)	329 (50.7%)	1,127 (41.7%)
**Alcoholic drinks/day**					
0–1 drink	393 (32.6%)	144 (31.9%)	116 (29.1%)	140 (21.6%)	793 (29.3%)
2 drinks	165 (13.7%)	47 (10.4%)	45 (11.3%)	82 (12.6%)	339 (12.5%)
3 or more drinks	59 (4.9%)	30 (6.7%)	32 (8.0%)	79 (12.2%)	200 (7.4%)
Depressive symptoms	3.3 (4.8)	3.3 (5.5)	3.9 (5.4)	3.7 (5.2)	3.5 (5.1)
Physical activity (hours/week)	2.0 (5.9)	2.4 (7.6)	3.8 (33.8)	2.3 (9.2)	2.4 (14.6)
Total cholesterol (mg/dl)	192.2 (42.6)	188.0 (44.2)	190.6 (43.6)	188.5 (42.5)	190.4 (43.0)
Systolic blood pressure (mmHg)	123.9 (19.4)	125.4 (20.6)	123.5 (19.8)	125.7 (20.3)	124.5 (19.9)
CERAD W-L immediate recall	19.0 (5.0)	18.3 (5.2)	18.4 (4.9)	18.0 (5.1)	18.6 (5.1)
CERAD W-L delayed recall	6.0 (2.4)	5.6 (2.6)	5.8 (2.4)	5.6 (2.3)	5.8 (2.4)
Animal fluency test	17.1 (5.5)	16.6 (6.0)	16.3 (5.1)	15.3 (5.4)	16.5 (5.5)
Digit symbol substitution test	49.2 (17.4)	45.9 (17.5)	45.8 (18.2)	42.2 (16.4)	46.5 (17.5)

^a^Data was presented as mean (standard deviation) for continuous variables and *n* (%) for categorical variables.

The means and 95% CI of the cognitive test-specific *z* scores by serum cotinine level quartiles were presented in Table 2. For participants in 1st quartile (lowest) of serum cotinine level, their mean *z* score of CERAD W-L immediate recall, CERAD W-L delayed recall, AFT, and DSST was 0.09 (95% CI = −1.85, 2.03), 0.10 (95% CI = −1.85, 2.05), 0.13 (95% CI = −1.81, 2.08), and 0.21 (95% CI = −1.75, 2.17), respectively. For participants in 2nd quartile of serum cotinine level, their mean *z* score of CERAD W-L immediate recall, CERAD W-L delayed recall, AFT, and DSST was −0.03 (95% CI = −2.07, 2.01), −0.08 (95% CI = −2.20, 2.04), 0.04 (95% CI = −2.08, 2.16), and 0.03 (95% CI = −1.94, 1.99), respectively. Among participants in the 3rd quartile, their mean *z* score of CERAD W-L immediate recall, CERAD W-L delayed recall, AFT, and DSST was 0.00 (95% CI = −1.92, 1.91), 0.03 (95% CI = −1.92, 1.97), −0.01 (95% CI = −1.81, 1.79), and 0.02 (95% CI = −2.02, 2.06), respectively. The mean *z* score of CERAD W-L immediate recall, CERAD W-L delayed recall, AFT, and DSST was −0.09 (95% CI = −2.10,1.92), −0.05 (95% CI = −1.98, 1.88), −0.18 (95% CI = −2.07,1.71), −0.19 (95% CI = −2.07, 1.65), respectively among participants in the 4th quartile (highest). The mean global cognition *z* score of quartiles 1–4 was 0.13 (95% CI = −1.82, 2.07), −0.04 (95% CI = −2.12, 2.04), −0.03 (95% CI = −1.93, 1.88), −0.21 (95% CI = −2.07, 1.65), respectively.

**TABLE 2:** Cognitive *z*-scores and 95% confidence intervals (CIs) by quartile of serum cotinine level.

	Quartile 1 ≤0.01 ng/ml	Quartile 2 0.01 < cotinine ≤ 0.02 ng/ml	Quartile 3 0.02 < cotinine ≤ 0.04 ng/ml	Quartile 4 >0.04 ng/ml
CERAD W-L immediate recall	0.09 (−1.85, 2.03)	−0.03 (−2.07, 2.01)	0.00 (−1.92, 1.91)	−0.09 (−2.10, 1.92)
CERAD W-L delayed recall	0.10 (−1.85, 2.05)	−0.08 (−2.20, 2.04)	0.03 (−1.92, 1.97)	−0.05 (−1.98, 1.88)
Animal fluency test	0.13 (−1.81, 2.08)	0.04 (−2.08, 2.16)	−0.01 (−1.81, 1.79)	−0.18 (−2.07, 1.71)
Digit symbol substitution test	0.21 (−1.75, 2.17)	0.03 (−1.94, 1.99)	0.02 (−2.02, 2.06)	−0.19 (−2.03, 1.66)
Global cognition	0.13 (−1.82, 2.07)	−0.04 (−2.12, 2.04)	−0.03 (−1.93, 1.88)	−0.21 (−2.07, 1.65)

Multivariate linear regressions results (Table 3) showed that participants in the 4th quartile (highest) of serum cotinine level, compared with those in the 1st quantile (lowest), had lower immediate recall (β = −0.16, 95% CI = −0.29, −0.03), AFT (β = −0.19, 95% CI = −0.33, −0.05), DSST (β = −0.27, 95% CI = −0.39, −0.15), and global cognition (β = −0.26, 95% CI = −0.39, −0.14) *z* scores. Participants in the 3rd quartile, compared with those in the 1st quartile, had lower immediate recall (β = −0.16, 95% CI = −0.30, −0.02) and global cognition (β = −0.16, 95% CI = −0.29, −0.02) *z* scores. Participants in the 2nd quartile, compared with those in the 1st quartile, had lower delayed recall *z* scores (β = −0.16, 95% CI = −0.29, −0.02). All results were controlled for age, race/ethnicity, education, depressive symptoms, body mass index, alcohol use, smoking history, prevalent CHD, stroke, and systolic blood pressure.

**TABLE 3:** The associations of quartile of serum cotinine level (Reference: ≤0.01 ng/ml) with test specific and global cognition *z* scores^a^.

	Quartile 1 ≤0.01 ng/ml	Quartile 2 0.01 < cotinine ≤ 0.02 ng/ml	Quartile 3 0.02 < cotinine ≤ 0.04 ng/ml	Quartile 4 >0.04 ng/ml
CERAD W-L immediate recall	Reference	−0.07 (−0.20, 0.07)	−**0.16 (**−**0.30,** −**0.02)**	−**0.16 (**−**0.29,** −**0.03)**
CERAD W-L delayed recall	Reference	−**0.16 (**−**0.29,** −**0.02)**	−0.14 (−0.28, 0.01)	−0.12 (−0.25, 0.01)
Animal fluency test	Reference	0.04 (−0.11, 0.19)	−0.02 (−0.18, 0.13)	−**0.19 (**−**0.33,** −**0.05)**
Digit symbol substitution test	Reference	−0.10 (−0.23, 0.02)	−0.10 (−0.23, 0.03)	−**0.27 (**−**0.39,** −**0.15)**
Global cognition	Reference	−0.10 (−0.22, 0.04)	−**0.16 (**−**0.29,** −**0.02)**	−**0.26 (**−**0.39,** −**0.14)**

^a^All models were adjusted for age (years), sex (male or female), race/ethnicity (Mexican Americans, other Hispanics, non-Hispanic Whites, or non-Hispanic Blacks), education (below high school, high school graduate, or some college or above), BMI (<18.5, 18.5–24.9, 25.0–29.9, or ≥30 kg/m^2^), alcohol use (0–1, 2, 3, or more drinks per day), smoking history (never or former), depressive symptoms, physical activity (hours/week), total cholesterol (mg/dl), and systolic blood pressure (mmHg). Bolded values mean statistically significant results (95% confidence interval excluding zero).

## Discussion

In this group of 2,703 US non-smoking older adults, higher serum cotinine level is independently associated with worse new verbal information learning ability, language proficiency, executive functioning, processing speed, sustained attention, working memory, as well as global cognition. Although our results still need to be validated by longitudinal studies, they indicate that prevention and reduction of SHS in older adults may help protect their cognitive functioning.

Controversy exists on the association between SHS and cognitive functioning in older adults, although a limited number of studies have examined the relationship between the two and found a negative cognitive effect of SHS. In a longitudinal study of 6,875 middle-aged and older Chinese women, women who had lived with a smoking husband experienced a significantly faster decline in global cognitive function, visuospatial ability, and episodic memory function than those who had not (Bai et al., 2020). However, in that study, researchers failed to adjust important covariates such as body mass index, exercise, and alcohol use. In addition, SHS was assessed by self-report. Based on findings of another longitudinal study of 970 participants, in comparison to participants with 0–15 years’ SHS exposure and ≤25% carotid artery stenosis, those with >25 years of SHS exposure and >25% carotid artery stenosis had a threefold increased dementia risk (Barnes et al., 2010). In that study, SHS was also assessed by self-report. In another study of college students (*n* = 68), daily prospective memory and executive function deficits were linked to SHS (Heffernan and O’Neill, 2013). However, the sample size of that study is small, and the researchers did not control any covariates. In another cohort study of Chinese middle-aged women, researchers found a significant decline in cognitive abilities, including memory, among Chinese women who are exposed to SHS, and this decline can last for up to 2 years (Pan et al., 2018). However, in that study, SHS was assessed by self-report, and the participants were not exclusively older adults. SHS was also assessed by self-report in another two relevant studies targeting older adults (Orsitto et al., 2012; He et al., 2020). In a cross-sectional study of 2,542 non-smoking older adults, each unit increase in log-transformed blood cotinine level resulted in a decrease in cognitive performance score of 2.03 points (Akhtar et al., 2013). However, the data of that study were collected two decades ago (between 1999 and 2002), and only one cognitive-specific test (the DSST) was analyzed. Thus, although many studies have examined this relationship, few used biomarkers to assess SHS and exclusively target a large sample of older adults.

The possible mechanisms that account for the negative association between SHS exposure and cognitive performance are complicated. One popular hypothesis is that the carbon monoxide in tobacco smoke obstructs blood’s delivery of oxygen to the brain (Levy, 2015). Another possible explanation comes from animal studies. Studies have shown that animals exposed to various concentrations of harmful chemicals contained in tobacco smoke experienced a decline in neuronal mass in regions of the brain related to learning and memory (Bai et al., 2020). SHS exposure may also increase the risk of cardiovascular disease (Penn et al., 1994; Teo et al., 2006), which in turn elevates the risk of cognitive impairment and dementia (Otsuka et al., 2001; Newman et al., 2005). Even short-term SHS exposure has been shown to have detrimental effects on endothelial function and instantly impairs the cardiovascular system (Hachinski, 2007). Dysfunctional endothelial cells may lead to vasoconstriction, atherogenesis, and thrombosis, which may restrict blood supply to the brain. Additionally, SHS exposure is a risk factor for stroke (Bernal-Pacheco and Román, 2007), and the discrepancies in subclinical cerebrovascular disease may account for the different cognitive functions in late adulthood (Allwright et al., 2005). Despite these proposed mechanisms, the exact reasons underlying the differential effects of SHS on specific cognitive domains are still unclear and need to be further studied.

There are many strengths of this study. To our knowledge, there are few studies that examined the relationship between SHS using serum cotinine level and cognitive functioning, exclusively targeting a large sample of non-smoking older adults. Therefore, our study makes a unique contribution to the literature and adds strong evidence of the negative cognitive effect of SHS in older adults. With our sample size being relatively large and representative, our study has good generalizability. Active smokers were removed using two criteria to make sure that the participants of this study were not current smokers. Additionally, global cognition was calculated to evaluate an older adult’s overall cognition. Moreover, to lessen the possibility of residual confounding, a wide range of socio-demographic, lifestyle, mental health, and physical health covariates were adjusted. Studies have shown that with long-term cumulative damage, the consequence of smoking disproportionately affects older adults. Compared to non-smokers, older adults who are exposed to tobacco are more likely to suffer from common age-related diseases like diabetes, osteoporosis, cardiovascular disease, chronic kidney disease, and respiratory issues with worse management and outcomes (Burns, 2000). Thus, our study targeted a vulnerable population and an overlooked issue.

This study is subject to several limitations. First, this study is a cross-sectional design which prevents us from assessing the temporal relationship between SHS and cognitive functioning. Moreover, since cotinine has a short half-life (15–20 h), it only measures a person’s recent exposure to tobacco and does not reflect his/her long-term exposure to tobacco. In addition, the excluded people due to missing data and being current smokers (*n* = 769) and the included participants (*n* = 2,703) had several ethnic, mental health, and lifestyle differences. Therefore, selection bias is likely (Lu et al., 2022). Finally, using three cognitive performance tests, we may not have assessed all cognitive domains. Future students are expected to use longitudinal designs to examine the temporal relationship between serum cotinine level and other biomarkers of tobacco exposure with a longer half-life, such as 4-(Methylnitrosamino)-1-(3-pyridyl)-1-butanol (NNAL) (Goniewicz et al., 2011) and the full cognitive domains in non-smoking older adults, especially those from non-western countries.

The clinical implications of this study are as follows: In this study, we found an independent and negative relationship between serum cotinine level, a biomarker of tobacco exposure, and various domains of as well as global cognitive functioning in non-smoking older adults. Given the negative effects of SHS on cognitive functioning in older adults, policymakers should continually promote smoking-free policies and use social media and other educational approaches to inform the public of the negative effect of SHS. Clinicians and health educators should encourage older adults to prevent and reduce SHS exposure in clinical and community settings (Hovell and Hughes, 2009). For example, if an older adult has a partner who is a smoker, he should be given instructions on specific measures to reduce SHS. These joint efforts may help protect older adults’ cognitive functioning.

In conclusion, higher serum cotinine level is independently associated with worse cognitive functioning in non-smoking older adults. Prevention and reduction of SHS in older adults may help protect their cognitive functioning.

## Data availability statement

The data that support the findings of this study are openly available on the NHANES website and can be accessed at https://wwwn.cdc.gov/nchs/nhanes/Default.aspx.

## Ethics statement

The NHANES were ethically reviewed and approved by the National Center for Health Statistics Research Ethics Review Board. The patients/participants provided their written informed consent to participate in this study.

## Author contributions

GQ, ZF, SG, and XL drafted the initial manuscript, designed the study, and searched for literature. ZQ and LX conducted statistical analysis. BS, LX, and FD critically revised the manuscript. All authors contributed to the article and approved the submitted version.
