# Supplementary material for: Higher blood cotinine level is associated with worse cognitive functioning in non-smoking older adults
Source: Front Neurosci. 2022 Nov 24;16:1080066. doi: 10.3389/fnins.2022.1080066 (PMC9730528; doi:10.3389/fnins.2022.1080066)
Supplement: Supplementary file 1 [file Table_1.DOCX]

Appendix. The characteristics of included (n=2703) and excluded participants (n=769)

| Variables | Excluded  (n=769) | Included (n=2703) | *P*-Value |
| --- | --- | --- | --- |
| Age, years | 68.2(6.5) | 70.5(7.0) | **<0.001** |
| Sex, n (%) |  |  | **<0.001** |
| Male | 434(56.4%) | 1253(46.4%) |  |
| Female | 335(43.6%) | 1450(53.6%) |  |
| Race/ethnicity, n (%) |  |  | **<0.001** |
| Mexican Americans | 76(9.9%) | 249(9.2%) |  |
| Other Hispanics | 75(9.8%) | 271(10.0%) |  |
| Non-Hispanic Whites | 275(35.8%) | 1305(48.3%) |  |
| Non-Hispanic Blacks | 279(36.3%) | 558(20.6%) |  |
| Other | 64(8.3%) | 320(11.8%) |  |
| Education, n (%) |  |  | **<0.001** |
| Below high school | 295(38.3%) | 723(26.7%) |  |
| High school graduate | 176(22.9%) | 621(23.0%) |  |
| Some college or above | 295(38.3%) | 1355(50.2%) |  |
| Body mass index, n (%) |  |  | **<0.001** |
| <18.5 kg/m2 | 30(3.9%) | 31(1.1%) |  |
| 18.5-24.9 kg/m2 | 259(33.7%) | 637(23.6%) |  |
| 25.0-29.9 kg/m2 | 234(30.4%) | 956(35.4%) |  |
| ≥30 kg/m2 | 219(28.5%) | 1029(38.1%) |  |
| Smoking |  |  | **<0.001** |
| Never | 164(21.3%) | 1573(58.2%) |  |
| Former | 161(20.9%) | 1127(41.7%) |  |
| Current | 444(57.7%) | - |  |
| Alcoholic drinks/day |  |  | **<0.001** |
| 0-1 drink | 69(9.0%) | 793(29.3%) |  |
| 2 drinks | 58(7.5%) | 339(12.5%) |  |
| 3 or more drinks | 64(8.3%) | 200(7.4%) |  |
| Depressive symptoms | 4.1(5.1) | 3.5(5.1) | **0.008** |
| Physical activity, hours/week | 2.1(6.8) | 2.4(14.6) | 0.591 |
| Total cholesterol, mg/dL | 190.742.9) | 190.4(43.0) | 0.888 |
| Systolic blood pressure, mmHg | 127.0(21.7) | 124.5(19.9) | **0.006** |
| CERAD W-L immediate recall | 18.1(4.9) | 18.6(5.1) | 0.053 |
| CERAD W-L delayed recall | 5.5(2.3) | 5.8(2.4) | **0.004** |
| Animal Fluency Test | 15.8(5.6) | 16.5(5.5) | **0.006** |
| Digit Symbol Substitution Test | 41.4(16.4) | 46.5(17.5) | **<0.001** |
